# Protective Effect of Tea Polyphenol Ophthalmic Gel on Lens Epithelial Cells in Rabbits with Silicone Oil Tamponade after Vitrectomy

**DOI:** 10.1155/2014/832381

**Published:** 2014-11-20

**Authors:** Xianzhen Ma, Dadong Guo, Hongsheng Bi, Xiaofeng Xie, Junguo Guo, Yan Cui

**Affiliations:** ^1^Affiliated Eye Hospital of Shandong University of Traditional Chinese Medicine, No. 48, Yingxiongshan Road, Jinan 250002, China; ^2^Shandong Provincial Key Laboratory of Integrated Traditional Chinese and Western Medicine for Prevention and Therapy of Ocular Diseases, Key Laboratory of Integrated Traditional Chinese and Western Medicine for Prevention and Therapy of Ocular Diseases in Universities of Shandong, Eye Institute of Shandong University of Traditional Chinese Medicine, No. 48, Yingxiongshan Road, Jinan 250002, China

## Abstract

*Purpose*. The aim of this study was to investigate the effect of tea polyphenols (TP) ophthalmic gel on lens epithelial cells (LECs) in rabbits with silicone oil tamponade after vitrectomy. *Methods*. In this study, unilateral vitrectomy with silicone oil tamponade was performed using 2-month-old New Zealand white rabbits (*n* = 72); meanwhile, age-matched nonoperated rabbits (*n* = 18) were used as controls. The TP ophthalmic gel was administered topically in the surgical eyes till they were sacrificed. On days 45 and 90 after operation, the levels of reactive oxygen species (ROS), mitochondrial membrane potential (ΔΨm), and apoptosis of LECs were analyzed, respectively. Meanwhile, caspase-3 mRNA and protein levels were also determined. *Results*. The results indicate that the levels of ROS and apoptosis were elevated for LECs in rabbits after operation, whereas ΔΨm was decreased. Caspase-3 was apparently increased at both mRNA and protein levels. Treatment of TP ophthalmic gel could reduce the generation of ROS, maintain ΔΨm, inhibit the overexpression of caspase-3, and thus decrease the apoptosis of LECs of rabbits after operation. *Conclusions*. TP ophthalmic gel can efficiently inhibit caspase-3 overexpression, reduce the apoptosis of LECs, and prevent LECs from damage. Our result provides a new approach to prevent the development of complicated cataract after vitrectomy.

## 1. Introduction

Vitrectomy is the surgical removal of the vitreous which can help treat retinal detachments and many other conditions. Silicone oil is frequently used in vitrectomy. However, the complicated cataract is a frequent complication after vitreoretinal surgery, and the rate is about 60%–98% for phakic eyes within 2 years [1, 2]. Vitrectomy surgery can significantly increase intraocular oxygen tension during and for prolonged periods after surgery, which may lead to nuclear cataract formation [3]. Meanwhile, vitrectomy with silicone oil tamponade can produce a characteristic transient posterior subcapsular cataract in the immediate postoperative period, and the acute alterations were followed by accelerated nuclear opacification [4]. Thus, this kind of cataract is an important reason for the decrease of visual acuity again after vitrectomy. Currently, there is no ideal medicine which can effectively prevent the cataractogenesis after vitrectomy.

Lens epithelial cells (LECs) are the most active parts of the lens metabolism. Their functional status is very important to maintain lens transparency [5, 6]. The apoptosis of LECs is involved in many factors including ultraviolet B irradiation [7–9] and exposure to nanomaterials [10]. In the meantime, researchers have confirmed that LEC apoptosis is a common cellular basis for noncongenital cataract development in human and animals [5, 11].

Traditional Chinese medicine is well-known for its various roles in preventing and treating infectious and chronic diseases; it differs from Western medicine because that traditional Chinese medicine can exhibit complicated bioactive components, triggering many signaling pathways and extensive actions on vital organs, and currently, modern scientific approaches have demonstrated some of their underlying mechanisms [12]. The advantages of traditional Chinese medicine can be summarized as follows: preventing tumorigenesis; reducing toxicity and enhancing the treatment effect; and attenuating tumor recurrence and metastasis [13]. Meanwhile, traditional Chinese medicine may also act as gene therapy vehicles, therapeutic genes, synergistic therapeutic treatments, and coadministrated drugs to reduce side effects [14]. Thus, traditional Chinese medicine has played roles in preventing and treating infectious and chronic diseases.

Tea polyphenols (TP) are tea extract ingredients including catechins, flavonoids, and phenolic acids. They are very good antioxidants and have powerful ability to scavenge free radicals [15–17]. TP can maintain stable mitochondrial membrane potential (ΔΨm) of target cells and decrease the LEC death rate under high glucose [18]. To investigate the protective effect of TP on LECs, we developed TP ophthalmic gel and explored the effect on the alterations in the levels of LEC apoptosis, ΔΨm, reactive oxygen species (ROS), and caspase-3 at gene and protein levels after vitrectomy with silicone oil tamponade. Further, we observed the protective effect of TP ophthalmic gel on LECs. The results indicate that TP ophthalmic gel could maintain stable ΔΨm, reduce the generation of ROS, and prevent LECs from apoptosis, indicating that TP ophthalmic gel possesses great potential in clinical practice to prevent the LEC apoptosis after vitrectomy with silicone oil tamponade.

## 2. Materials and Methods

### 2.1. Principal Component Analysis of TP

Tea polyphenols were purchased from Shanxi Sciphar Hi-tech Industry Co., Ltd. The standard reference substances (gallic acid, catechin, gallocatechin, epigallocatechin gallate, gallocatechin gallate, and epicatechin gallate) were supported by National Institutes for Food and Drug Control, China. Principal component analysis of TP was performed by high performance liquid chromatography (HPLC) (Ultimate 3000, Dionex, Thermo Scientific, USA) with a DIONEX Acclaim-C18 (4.6 mm × 250 mm, 5 *μ*m) column. The HPLC conditions were mobile phase A: methanol-0.1% formic acid (10 : 90) and mobile phase B: methanol-acetonitrile-0.1% formic acid. The linear elution procedure was 0 min (0% B)-10 min (10% B)-20 min (20% B)-40 min (30% B)-50 min (50% B)-60 min (10% B). The wavelength was 280 nm and the flow rate was 1.0 mL/min.

### 2.2. Preparation of TP Ophthalmic Gel

Poloxamers are polyoxyethylene-polypropylene block copolymer nonionic surfactants [19] which include hydrophilic corona ethylene oxide and hydrophobic core (polypropylene oxide) blocks arranged in a triblock structure resulting in an amphiphilic copolymer [20].

The preparation of TP ophthalmic gel was performed as follows: the poloxamer P188 (Sigma-Aldrich, St. Louis, MO, U.S.A.) solution (5%) was prepared in distilled water, and then poloxamer P407 (Sigma-Aldrich, St. Louis, MO, U.S.A.) was added under mixing condition till it was fully dissolved and the final concentration of poloxamer P407 was 25%. Further, the mixed solutions were stored at 4°C overnight and further were mixed with 0.1% carbopol (Noveon Inc., U.S.A.) solution. Finally, TP were added and were stirred till they were completely dissolved in the mixed solutions. The concentration of TP was 5% (w/v). Meanwhile, ophthalmic gel without TP (control gel) was also prepared in the same way.

### 2.3. Experimental Animals and Administration

New Zealand white rabbits (1.0–1.5 kg, 2-month-old) were purchased from Animal Center of Shandong University of Traditional Chinese Medicine. All experimental procedures adhered to the Association for Research in Vision and Ophthalmology Statement for the use of animals in ophthalmic and vision research. The protocol was approved by the Committee on the Ethics of Animal Experiments of Shandong University of Traditional Chinese Medicine. Prior to the experiment, the rabbits were checked with a slit lamp microscope (Topcon, Japan) and an indirect ophthalmoscope (Wechallyn, USA). The animals with transparent lenses and without other eye diseases were used in the experiments.

Ninety rabbits were used in this study and were randomly divided into three groups according to the table of random numbers: normal control group (*n* = 18), model group (*n* = 36), and treatment group (*n* = 36). The rabbits in normal control group were untreated. Unilateral vitrectomy (DORC, Zuidland, the Netherlands) with silicone oil (Bausch & Lomb, U.S.A) tamponade was performed both in model and in treatment rabbits. The samples with cataract formed by mechanical damage using vitrectomy were excluded. After operation, the ophthalmic gel with or without TP (0.5%) was administered topically to the surgical eyes in treatment (0.5% TP ophthalmic gel) or model groups (ophthalmic gel without TP) till the rabbits were sacrificed. All rabbits were administered 4 times a day.

### 2.4. Vitrectomy and Cataract Development

Vitrectomy with silicone oil tamponade was performed according to the literature [21]. Eyes were examined after surgery by a slit-lamp microscope and an indirect ophthalmoscope to monitor the retinal hemorrhage, retinal detachment, or traumatic cataract. The lenses of 18 rabbits in each group were observed and were taken photographs by a slit-lamp microscope with photograph equipment on days 15, 30, 45, 60, and 90 after vitrectomy. The number of the rabbits with lens opacification was recorded.

### 2.5. Preparation of LECs

On days 45 and 90 after vitrectomy, rabbits (*n* = 18) were sacrificed and the eyes were carefully excised. After eyes were rinsed with 75% ethanol for 30 s, lenses were carefully dissected and were washed in phosphate buffered saline solution (PBS) three times to remove attached pigments and vitreous. Lens capsule epithelial cell samples were collected by curvilinear continuous capsulorhexis (CCCE) in a 35 mm^2^ culture dish with 1 mL PBS and were further cut into pieces with fine scissors and further were trypsinized with 400 *μ*L 0.25% trypsin for 5 min in ice bath. The trypsinization was terminated with 400 *μ*L Dulbecco's Modified Eagle's Medium (DMEM, Life technologies, USA) containing 10% bovine serum (HyClone, USA). All samples were filtered with 200 mesh sieve and the cell suspensions were centrifuged (4°C, 1300 g) for 5 min and the supernatants were discarded. The pellets were washed with PBS twice and were finally suspended in PBS.

### 2.6. Measurement of Intracellular ROS

The level of intracellular ROS was measured using a ROS detection kit (Beyotime, China). Briefly, cells from pooled samples were incubated with 2′-7′-dichlorofluorescein-diacetate (DCFH-DA) solution (Beyotime, China) in the dark at 37°C for 30 min and then were washed with PBS and analyzed using a flow cytometer (Accuri C6, USA) within 30 min. The level of ROS was expressed as the percentage of fluorescent cells (fluorescent cells/total cells × 100%) and the experiment was repeated three times.

### 2.7. Analysis of ΔΨm (JC-1 Staining)

The changes in ΔΨm were explored using ΔΨm detection kit (Beyotime, China), in which the 5,5′,6,6′-tetrachloro-1,1′,3,3′-tetraethyl benzimidazolyl carbocyanine iodide (JC-1) was considered as the probe. ΔΨm is an important parameter of mitochondrial function that is used as an indicator of cell apoptosis [22, 23]. JC-1 can selectively enter the mitochondria and reversibly change color from red to green when membrane potential decreased. Briefly, cells from pooled samples were washed with PBS and incubated with JC-1 staining working solution at 37°C for 20 min, followed by resuspension in staining buffer solution. After staining, data acquisition and analysis were performed immediately by a flow cytometer (Accuri C6, USA). ΔΨm was expressed as the ratio of red fluorescence and green fluorescence (FL2/FL1) in histogram. The experiment was repeated three times.

### 2.8. Determination of Apoptosis of LECs (Annexin V/PI Staining)

Annexin V-FITC/propidium iodide (PI) apoptosis detection kit (KeyGEN, China) was used to determine the apoptosis of LECs. Briefly, cells from pooled samples were washed with PBS and were resuspended in Annexin V-FITC binding buffer at a final cell concentration of 1 × 10^6^ cells/mL, and then cells were incubated with both Annexin V-FITC and PI for 15 min in the dark. After staining, cells were performed immediately by a flow cytometer (Accuri C6, USA). The level of LEC apoptosis was expressed as the percentage of Annexin V^+^ PI^−^ cells in total determined cells and the experiment was performed three times.

### 2.9. Caspase-3 mRNA Expressions

On days 45 and 90 after operation, rabbits were, respectively, sacrificed and the eyes were carefully excised and were rinsed with 75% ethanol for 30 s; further lenses were isolated and LECs were collected and stored at −80°C prior to use. Total RNA was isolated from individual lens epithelia using TRIzol reagent (Invitrogen) prior to reverse transcription using the RevertAid reverse transcriptase (Fermentas), as previously described [24]. After determination of RNA concentration by a spectrophotometer (K5600, Beijing Kaiao Technology Development Co., Ltd., China), real-time quantitative PCR was performed with SYBR Green Master Mix (Aidlab, China) on a Stratagene Mx3000P (Agilent Technologies, USA). The primer sequences used were as follows: caspase-3 forward: 5′-AGTCTGACTGGAAAGCCGAA-3′, reverse: 5′-CGGGATCTGTTTCTTTGCAT-3′; GAPDH forward: 5′-GTGCCCATCTACGAGGGTTA-3′, and reverse: 5′-TCTCAGCTGTGGTGGTGAAG-3′. The PCR program was set as follows: 95°C for 10 min, followed by 45 cycles of a 95°C denaturation for 30 s, 56°C annealing for 30 s, and 72°C extension for 30 s. The ΔΔct values were calculated as fold change after normalization to respective endogenous GAPDH. Each experiment was performed three times.

### 2.10. Immunohistochemical Analysis of Caspase-3

On days 45 and 90 after operation, rabbits were, respectively, sacrificed and the eyes were carefully excised and were rinsed with 75% ethanol for 30 s; then lenses were isolated and fixed in 10% formalin, embedded in the paraffin, and cut into sections. Caspase-3 was analyzed using immunohistochemical analysis followed by the manufacturer's protocol (Beyotime, China). Briefly, slides were exposed to 3% H_2_O_2_ for 10 min to block endogenous peroxidase and then were rinsed with PBS. After incubation with primary antibody at 4°C overnight, slides were washed with PBS for 5 min and incubated with secondary antibody at 37°C for 30 min, and then slides were visualized using DAB under an optical microscope (Nikon Ti, Japan). Caspase-3 positive cells were counted from 5 random high-power fields in each section; the level of caspase-3 protein was expressed as the percentage of positive cells (positive cells/total cells × 100%). The experiment was repeated three times.

### 2.11. Statistical Analysis

Data were expressed as mean ± S.D., and the statistical analysis was performed with *t* test using SPSS17.0 statistical software. *P* < 0.05 was considered as statistical significance.

## 3. Results

### 3.1. Epigallocatechin Gallate Is the Principal Component of Tea Polyphenols

**Figure 1:**
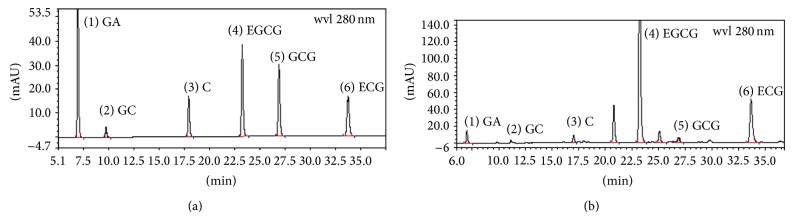
The chromatogram for principal component analysis of tea polyphenols based on HPLC using a DIONEX Acclaim-C18 (4.6 mm × 250 mm, 5 *μ*m) column. (a) Chromatogram of standard reference substances; (b) chromatogram of sample. In chromatogram, GA = gallic acid, EGCG = epigallocatechin gallate, GC = gallocatechin, C = catechin, GCG = gallocatechin gallate, and ECG = epicatechin gallate.

Using HPLC technique, we analyzed the principal components in TP. We found that epigallocatechin gallate was the main component in tea polyphenols and its content was 58.44% (Figure 1).

### 3.2. Lens Opacity Develops Seriously after Vitrectomy

**Figure 2:**
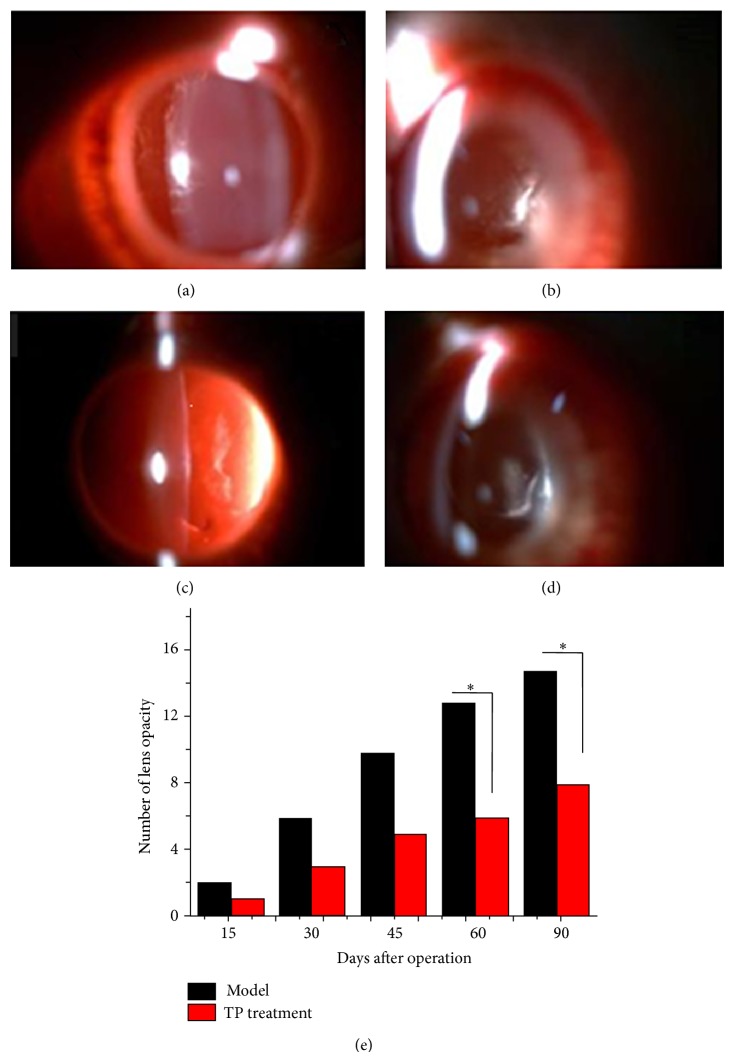
Effects of TP ophthalmic gel on cataract development after vitrectomy with silicone oil tamponade. The lenses of 18 rabbits in control, model, and treatment groups were observed and were taken photographs by a slit-lamp microscope on days 15, 30, 45, 60, and 90 after vitrectomy. Typical photographs showed the lens opacity: (a) model group, day 45; (b) model group, day 90; (c) treatment group, day 45; (d) treatment group, day 90. Meanwhile, there was significant difference between model group and treatment group (e). ^*^ *P* < 0.05.

Lenses in control group were transparent all along, yet lens opacity developed from day 15 after vitrectomy in model group and it became much more serious. However, the degree of lens opacity in treatment group was lower than in model group (Figure 2).

### 3.3. TP Ophthalmic Gel Reduces the Generation of Intracellular ROS

**Figure 3:**
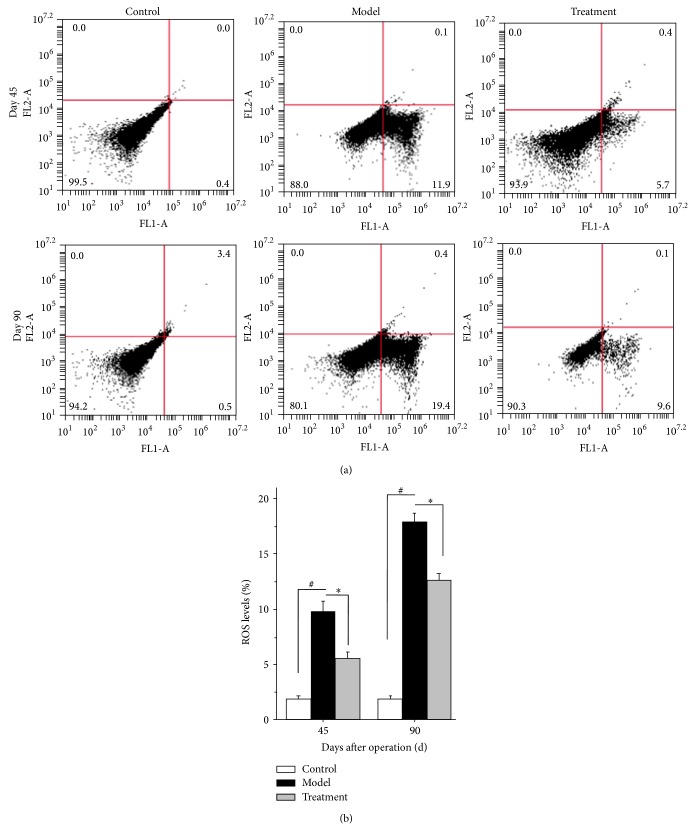
Effects of TP ophthalmic gel on the level of intracellular ROS of LECs in control, model, and treatment groups after operation. (a) ROS levels in LECs in control, model, and treatment groups after vitrectomy with silicone oil tamponade measured by flow cytometry either on day 45 or on day 90. (b) Histogram of ROS level of LECs in control, model, and treatment groups after operation. Data were from 3 independent experiments. ^#^ *P* and ^*^ *P* < 0.05.

The results indicate that vitrectomy with silicone oil tamponade treatment caused the elevated intracellular ROS in LECs. As shown in Figure 3, the levels of intracellular ROS were 1.8 ± 0.2%, 9.77 ± 0.96%, and 5.58 ± 0.57% for control, model, and treatment group on day 45, respectively. On day 90, they were 1.8 ± 0.3%, 17.93 ± 0.8%, and 12.6 ± 0.62%, respectively. The levels of intracellular ROS in the model group were markedly elevated compared with those in the relevant control group on days 45 and 90 after operation (*P* < 0.05). However, after treatment with TP ophthalmic gel, the levels of intracellular ROS in the treatment group were significantly decreased compared with those in model group both on day 45 and on day 90 (*P* < 0.05).

### 3.4. TP Ophthalmic Gel Restores the Levels of ΔΨm

**Figure 4:**
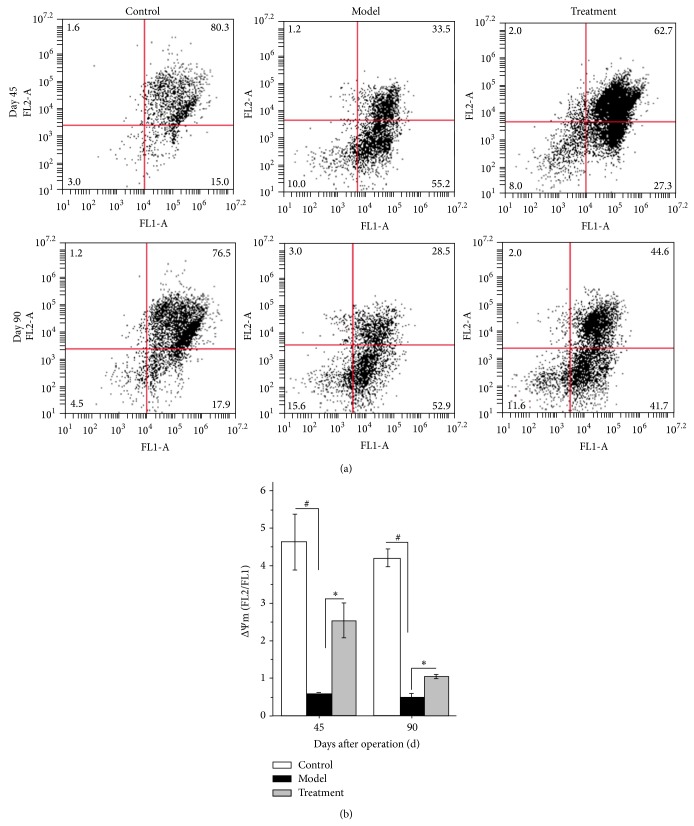
Effects of TP ophthalmic gel on mitochondrial membrane potential of LECs in control, model, and treatment groups after vitrectomy with silicone oil tamponade. (a) Mitochondrial membrane potential in LECs in control, model, and treatment groups after operation determined by flow cytometry either on day 45 or on day 90. (b) Histogram of membrane potential of LECs in control, model, and treatment groups after operation. Data were from 3 independent experiments. ^#^ *P* and ^*^ *P* < 0.05.

As shown in Figure 4, the levels of ΔΨm were 4.62 ± 0.74%, 0.6 ± 0.026%, and 2.53 ± 0.47% for control, model, and treatment group on day 45, respectively. On day 90, they were 4.21 ± 0.23%, 0.51 ± 0.08%, and 1.05 ± 0.15%, respectively. The levels of ΔΨm in the model group were markedly decreased compared with those in the control group on days 45 and 90 after operation (*P* < 0.05), and the level of ΔΨm on day 45 was apparently lower than that on day 90. However, after treatment with TP ophthalmic gel, the levels of ΔΨm in the treatment group were significantly increased compared with those in model group both on day 45 and on day 90 (*P* < 0.05).

### 3.5. TP Ophthalmic Gel Attenuates LEC Apoptosis

**Figure 5:**
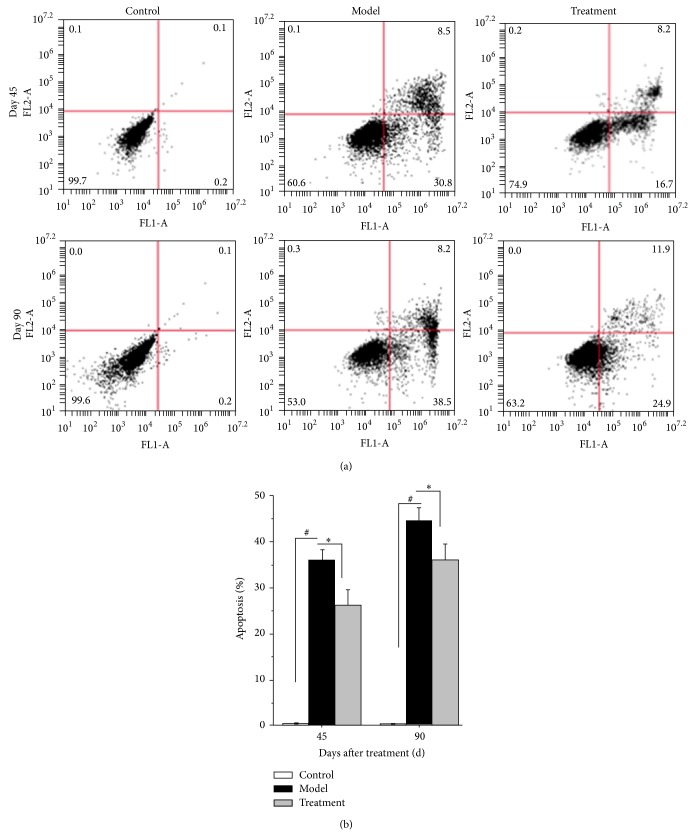
Effects of TP ophthalmic gel on the apoptosis of LECs in each group after operation. (a) Apoptotic levels of LECs in control, model, and treatment groups after operation determined by flow cytometry either on day 45 or on day 90. (b) Histogram of apoptotic levels of LECs in control, model, and treatment groups after operation. Data were from 3 independent experiments. ^#^ *P* and ^*^ *P* < 0.05.

To determine the effect of TP ophthalmic gel on LECs after operation, Annexin V/PI staining analyses were performed by flow cytometry. As shown in Figure 5, the percentages of Annexin V-FITC-positive cells were 0.4 ± 0.13%, 36.3 ± 1.88%, and 26.2 ± 3.42% for control, model, and treatment group on day 45 and were 0.3 ± 0.11%, 44.87 ± 2.65%, and 37.37 ± 3.38% on day 90, respectively. The levels of LEC apoptosis in model group were significantly more than in control groups on days 45 and 90 after operation (*P* < 0.05). However, after treatment with TP ophthalmic gel, the percentages of LEC apoptosis were apparently reduced.

### 3.6. TP Ophthalmic Gel Decreases the Expression of Caspase-3 mRNA

**Figure 6:**
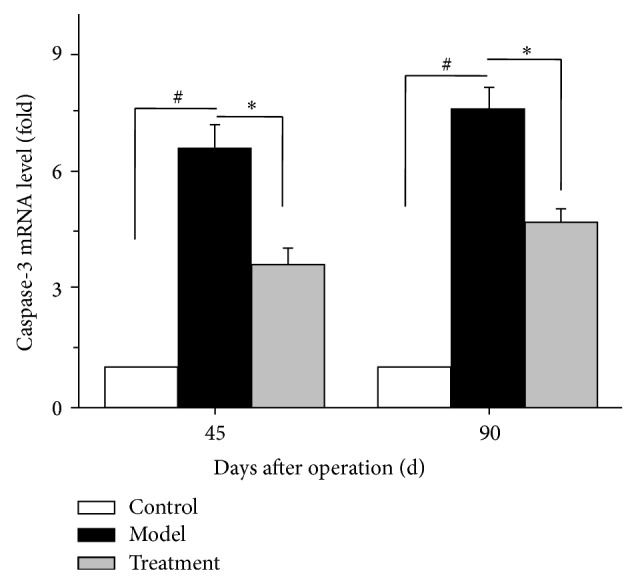
Effects of TP ophthalmic gel on caspase-3 mRNA expression of LECs in control, model, and TP ophthalmic gel groups after operation. Caspase-3 mRNA expression was detected by real-time quantitative PCR using SYRB Green I. Data were run in triplicate and results were represented as mean ± S.E. of three independent experiments. ^#^ *P* < 0.05 compared with control group and ^*^ *P* < 0.05 compared with model group.

As shown in Figure 6, the levels of caspase-3 mRNA in model group were increased to 6.57- and 7.6-fold on days 45 and 90 compared with the relevant control samples, respectively. However, after treatment with TP ophthalmic gel, the levels of caspase-3 mRNA were decreased to 3.6- and 4.67-fold on days 45 and 90, respectively. The levels of caspase-3 mRNA in treatment group were significantly decreased compared with those in the relevant model group (*P* < 0.05).

### 3.7. TP Ophthalmic Gel Reduces the Levels of Caspase-3 Protein

**Figure 7:**
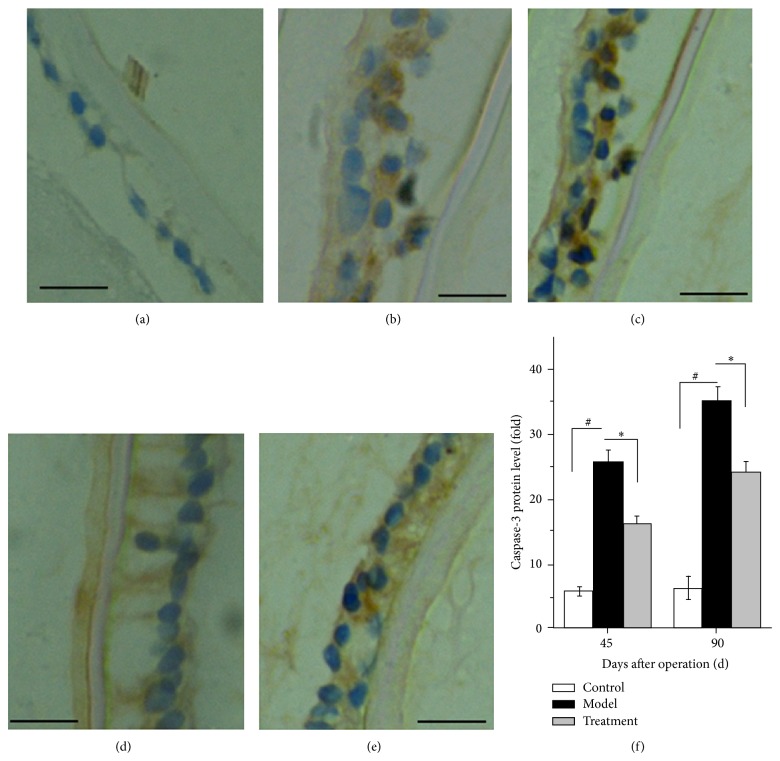
Effects of TP ophthalmic gel on caspase-3 expression of LECs in control, model, and treatment groups after operation determined by immunohistochemistry. (a) Control group; (b) model group, day 45; (c) model group, day 90; (d) treatment group, day 45; (e) treatment group, day 90; and (f) histogram of caspase-3 protein expression in each group. Data were from 3 independent experiments. ^#^ *P* < 0.05 compared with control group and ^*^ *P* < 0.05 compared with model group. Bar = 20 *μ*m.

Using immunohistochemistry analysis, the levels of caspase-3 protein of LECs in control, model, and treatment groups were determined after operation.  Figure 7 indicates that the percentages of caspase-3-protein-positive cells in model group were 5.8 ± 0.62%, 25.67 ± 1.74%, and 16.13 ± 1.21% for control, model, and treatment group on day 45, respectively; on day 90, they were 6.2 ± 1.83%, 34.95 ± 2.27%, and 24.03 ± 1.77%, respectively. The levels of caspase-3 in model group were significantly increased compared with those in control group after operation (*P* < 0.05). Importantly, the levels of caspase-3 protein in treatment group were significantly decreased compared with those in model group (*P* < 0.05).

## 4. Discussion

Vitrectomy is frequently used to treat the vitreoretinal disease. The complicated cataract is the most common complication after vitrectomy. It is reported that the development of complicated cataract after vitrectomy occurred about 60–98% of phakic eyes within 2 years [1, 2]. In 1962, silicone oil was used in retinal detachment surgery at the first time. After that, the silicone oil was widely used in vitreoretinal surgery.

Cataract is the most common complication after vitrectomy with silicone oil tamponade. Some scholars confirmed that cataracts developed in all phakic eyes with silicone oil tamponade after vitrectomy within 6 months [25]. Holekamp et al. reported that vitrectomy surgery can markedly increase intraocular oxygen tension during and for prolonged periods after surgery, and this exposes the crystalline lens to abnormally high oxygen and may lead to nuclear cataract formation [3]. Thus, decrease in reactive oxygen species may prevent cataract formation.

In our study, the development of complicated cataract after vitrectomy with silicone oil tamponade occurred in 77% of eyes on day 90 after operation, and it is consistent with the literature mentioned above. It was reported that some proteins, vitamin C, and lactic acid were found elevated in the vitreous cavity after vitrectomy, and all of them could scavenge active free radical and singlet oxygen and convert them into peroxide ions [26], indicating that the elevation of reactive oxygen species is involved in the process of complicated cataract. Studies have found that the oxidation of lens protein during vitrectomy was one of the reasons for nuclear cataract after vitrectomy [27], whereas adding antioxidants into the perfusion fluid during vitrectomy could inhibit the abnormal lens epithelial cell growth and lens fiber formation and thus prevent the cataractogenesis [28].

Lens is stretchable, avascular, and transparent organization. Lens epithelial cells are the most active parts of the lens metabolism; their functional status is very important to maintain lens transparency [5, 6]. Apoptosis is a highly regulated process of cell deletion and plays a fundamental role in the maintenance of tissue homeostasis in the normal organism. It is a physiological mode of cell death which is important in normal tissue development and remodeling [29]. LEC apoptosis is a common cellular basis for noncongenital cataract development in human and animals [5]. Some studies consider that apoptosis leads to lower densities of lens epithelial cells. It affected the lens fiber growth and growth quality [30–32]. In our studies, the Annexin V/PI staining has shown that the LEC apoptosis was apparently increased after vitrectomy with silicone oil tamponade, indicating that the operation can influence the normal physiological status of the cells.

It is well known that the disaggregation of the ΔΨm is a specific sign of apoptosis [33]. The main reason for the ΔΨm disaggregation is the mitochondrial permeability transition (PT). The occurrence of ΔΨm disaggregation is the direct consequence of the PT hole open. It can cause a series of important events related to apoptosis, one of which is the release of Ca^2+^ and other ions and proton leakage in mitochondria [34]. A series of specific cellular pathology phenomena such as Ca^2+^ overload, loss of ΔΨm, break of respiratory chain, and excess production of ROS will cause cell damage even death. Our findings demonstrate that vitrectomy with silicone oil tamponade could lead to the marked decrease of ΔΨm and increase of ROS production, which could aggravate LECs apoptosis.

Caspases are important signs of apoptosis [35, 36]. Several studies have shown that caspase-3 can be taken as a major downstream effector caspase in the apoptotic process, including apoptosis of lens epithelial cells [37–39]. In our studies, the mRNA and the protein levels of caspase-3 were elevated after vitrectomy with silicone oil tamponade, suggesting that caspase-3 is involved in apoptosis of LECs after operation.

TP are tea extract ingredients; their major components are four kinds of catechins including epigallocatechin gallocatechin gallate (EGCG), epicatechin gallate (ECG), epigallocatechin gallocatechin (EGC), and epicatechin (EC). They are easily oxidized because of their phenol-containing hydroxide radical. Therefore, they are very good antioxidant and have strong ability of free radical scavenging. The mechanism includes the following cases: (1) directly scavenging reactive oxygen radicals [16, 40, 41]: the phenol-containing hydroxide radicals in TP structure provide active hydrogen radicals to inactivate free radicals; (2) inhibition of lipid peroxidation: tea polyphenols can scavenge the intermediate product of lipid peroxyl radicals and alkoxyl radicals in the chain reaction, thereby preventing the propagation and amplification of lipid free radicals; (3) chelating metal ions: transition metals such as iron and copper are catalyst of free radical generation. The catechol structure in TP can chelate iron ions and form nonactive iron complexes, thus affecting oxidation process [15, 42]; (4) activation of the intracellular antioxidant defense system: tea polyphenols can hydrogenize the VE radicals and make VE regenerate and reduce the wear and tear of the VE [43].

In our previous study [18], we observed that TP solution can maintain stable mitochondrial membrane potential (ΔΨm) of target cells and decrease the LEC death rate under high glucose. To meet the requirements of clinical practice, we performed the TP ophthalmic gel and further investigated the effect of TP ophthalmic gel on the lens epithelial cells in rabbits with silicone oil tamponade after vitrectomy. In our studies, TP ophthalmic gel could efficiently reduce the generation of reactive oxygen species in LECs and alleviate ROS-induced damage to maintain normal structure and function of LECs. Furthermore, TP ophthalmic gel could efficiently inhibit the overexpression of caspase-3 and reduce the apoptosis of LECs and thus prevent LECs from death. Hence, the TP ophthalmic gel treatment provided a new strategy to decrease the generation of complicated cataract after vitrectomy.

## 5. Conclusions

In summary, the effect of TP ophthalmic gel on lens epithelial cells was investigated in rabbits after vitrectomy with silicone oil tamponade. The results indicate that TP ophthalmic gel could efficiently reduce the generation of reactive oxygen species, maintain the ΔΨm, inhibit the overproduction of caspase-3 both at gene and at protein levels, and thus prevent lens epithelial cells from apoptosis. TP ophthalmic gel has great potential in clinical practice in preventing the occurrence of the development of complicated cataract after vitrectomy with silicone oil tamponade.
